# Comparatively Evaluating the Role of Herb Pairs Containing Angelicae Sinensis Radix in Xin-Sheng-Hua Granule by Withdrawal Analysis

**DOI:** 10.1155/2020/9456350

**Published:** 2020-09-22

**Authors:** Han-Qing Pang, Ding-Qiao Xu, Yu-Ping Tang, Gui-Sheng Zhou, Hui-Qin Xu, Yi Jin, Zhen-Hua Zhu, Xu-Qin Shi, Shi-Jun Yue, Yan-Yan Chen, Sheng-Liang Huang, Jin-Ao Duan

**Affiliations:** ^1^Key Laboratory of Shaanxi Administration of Traditional Chinese Medicine for TCM C, and State Key Laboratory of Research & Development of Characteristic Qin Medicine Resources (Cultivation), and Shaanxi Key Laboratory of Chinese Medicine Fundamentals and New Drugs Research, and Shaanxi Collaborative Innovation Center of Chinese Medicinal Resources Industrialization, Shaanxi University of Chinese Medicine, Xi'an 712046, Shaanxi Province, China; ^2^Jiangsu Collaborative Innovation Center of Chinese Medicinal Resources Industrialization, and National and Local Collaborative Engineering Center of Chinese Medicinal Resources Industrialization and Formulae Innovative Medicine, and Jiangsu Key Laboratory for High Technology Research of TCM Formulae, Nanjing University of Chinese Medicine, Nanjing 210023, Jiangsu Province, China; ^3^Jiangsu Rongyu Pharmaceutical Co., Ltd., Huaian 223200, Jiangsu Province, China

## Abstract

The present study aims to investigate the roles of herb pairs containing Angelicae Sinensis Radix (Danggui) in Xin-Sheng-Hua Granule (XSHG) on hemolytic and aplastic anemia (HAA) mice. HAA model mice were induced by acetyl phenylhydrazine and cyclophosphamide; then the samples of XSHG and its decomposed recipes (DY, DC, DT, DH, DJ, and DZ) were orally administrated to these mice. Indicators of peripheral blood routine, organ index, and ATPase activities were tested. Moreover, the main effective components in these samples were also analyzed by UHPLC-TQ-MS/MS. Clear separation between the control and model groups from score plot of principal component analysis (PCA) was easily seen, indicating that HAA model was successfully conducted. Afterwards, relative distance calculation method between dose groups and control group from PCA score plot was adopted to evaluate the integrated effects of hematinic function of different samples. And the orders of hematinic effects were as follows: XHSG > DJ > DT > DZ > DH > DC > DY. Further analysis of these samples by UHPLC-TQ-MS/MS revealed that XSHG underwent complicated changes when herb pairs containing Danggui were excluded from XSHG, respectively. Compared with XSHG, the vast majority of active compounds in sample DY (formula minus herb pair Danggui-Yimucao) decreased significantly, which could partly explain why herb pair Danggui-Yimucao made great contribution to XSHG. These findings showed that withdrawal analysis method is a valuable tool to analyze the impacts of herb pairs containing Danggui on XSHG, which could lay foundation to reveal the compatibility rules of this formula.

## 1. Introduction

Traditional Chinese medicines (TCMs) have made tremendous contributions to the health of the ancient Chinese people for a long time. To achieve maximum therapeutic effect of certain diseases, TCMs are usually used in combination with several specific herbs instead of single herbs [1]. Xin-Sheng-Hua Granule (XSHG), originating from the classic and typical formulae Sheng-Hua Decoction, has function of promoting blood circulation, nourishing blood, and relieving pains [2]. And the formula is the boiled water extract of seven medical herbs, Angelicae Sinensis Radix (Danggui, DG), Leonuri Herba (Yimucao, YMC), Chuanxiong Rhizoma (Chuanxiong, CX), Persicae Semen (Taoren, TR), Carthami Flos (Honghua, HH), Cirsii Zingiberis Rhizoma Carbonisatum (Jiangtan, JT), and Glycyrrhizae Radix Et Rhizoma Praeparata Cum Mell (Zhigancao, ZGC), at a proportion of 80 : 90 : 30 : 8 : 5 : 5 : 5 [3]. According to the basic theory of TCM, XSHG is usually clinically applied to treat postpartum diseases including uterine hemorrhage, postpartum abdominal pain, and retention of the lochia after delivery [4]. And it is well known that postpartum diseases are usually associated with blood deficiency. Therefore, studying the effects of nourishing blood of XSHG is significant for researchers to discover its action mechanism.

In ancient China, two certain herbs are frequently used together in many formulae for the treatment of several diseases, which are also called herb pairs, *Yaodui* or *Duiyao*. They usually could achieve the effects of assistance, restraint, suppression, mutual antagonism, or mutual enhancement. As the fundamental composition units of TCM formulae, herb pairs are significant for researchers to study the compatibility rules of complicated formulae [5]. Interestingly, a series of herb pairs containing Danggui could be formed when Danggui was combined with other six single herbs from XSHG, respectively, including herb pairs Danggui-Yimucao, Danggui-Chuanxiong, Danggui-Taoren, Danggui-Honghua, Danggui-Jiangtan, and Danggui-Zhigancao. Ever since a long time ago, herb pairs including Danggui have usually been applied clinically for the treatment of women's diseases [6]. Based on our previous studies, the synergistic and complementary effects of promoting blood circulation and nourishing blood could be discovered in herb pairs Danggui-Chuanxiong and Danggui-Honghua [7, 8]. Undoubtedly, herb pairs containing Danggui made extremely significant contribution to this formula. In order to disclose the compatibility rules of XSHG from the aspects of herb pairs, it has become particularly important for researchers to investigate the contribution of these herb pairs to XSHG. As one of the decomposed analysis methods, the withdraw analysis has been applied to explore the compatibility rules of multiherb formulae due to the advantages of simplifying the complexity [9], suggesting that this method could be effectively applied to study the compatibility rules of XSHG.

To investigate the roles of herb pairs containing Danggui in the formulae on hemolytic and aplastic anemia (HAA) mice, the sample XSHG and six different samples of decomposed recipes were prepared separately, including sample DY (the formulae minus herb pair Danggui-Yimucao), DC (minus Danggui-Chuanxiong), DT (minus Danggui-Taoren), DH (minus Danggui-Honghua), DJ (minus Danggui-Jiangtan), and DZ (minus Danggui-Zhigancao). Then principal component analysis (PCA) model was applied to evaluate integrated effects of hematinic function of XSHG and these decomposed recipes [10]. Additionally, twenty-one bioactive constituents in these samples [11–15], including five phthalides, four aromatic acids, three alkaloids, four flavonoids, three gingerols, and two other components, were also determined simultaneously by ultra-high-performance liquid chromatography coupled with a triple quadrupole electrospray tandem mass spectrometry (UHPLC-TQ-MS/MS). And the content variations of active components were further compared between the formulae and the decomposed recipes in order to discover potential reasons of their effects.

In this study, the withdrawal analysis approach was originally applied to study the compatibility rules of XSHG from the aspects of herb pairs. Besides, a sensitive, reliable, and powerful technique capable of quantifying 21 marker components in XSHG was firstly proposed and validated by UHPLC-TQ-MS/MS method within 20 min. According to the above analysis, the contribution of these herb pairs to the formula XSHG was systematically investigated to discover their action characteristics in XSHG. Furthermore, our findings could lay foundation to further reveal the compatibility rules of XSHG.

## 2. Materials and Methods

### 2.1. Chemicals and Reagents

The herbs were generously provided by Revolence Pharmaceutical Co., Ltd. (Jiangsu, China), in February 2016, including *Angelica sinensis* (Oliv.) Diels, *Leonurus japonicus* Houtt., *Ligusticum chuanxiong* Hort., *Prunus persica* (L.) Batsch, *Carthamus tinctorius* L., roasted rhizome of *Zingiber* officinale Rosc, and Radix Glycyrrhizae Preparata; detailed information is summarized in Table S1. The seven herbs were kindly identified by Dr. Qinan Wu, doctor of pharmacognosy (College of pharmacy, Nanjing University of Chinese Medicine, Nanjing, China), and the voucher specimens were deposited at the Herbarium of Nanjing University of Chinese Medicine.

**Figure 1:**
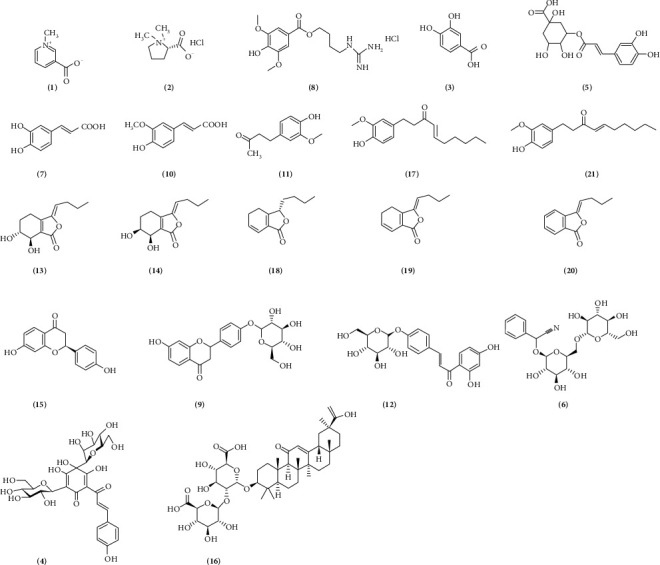
Chemical structures of 21 reference compounds of XSHG (1, trigonelline; 2, stachydrine hydrochloride; 3, protocatechuic acid; 4, hydroxysafflor yellow A; 5, chlorogenic acid; 6, amygdalin; 7, caffeic acid; 8, leonurine hydrochloride; 9, liquiritin; 10, ferulic acid; 11, zingerone; 12, isoliquiritoside; 13, senkyunolide I; 14, senkyunolide H; 15, liquiritigenin; 16, glycyrrhizic acid; 17, 6-gingerol; 18, senkyunolide A; 19, ligustilide; 20, butylidenephthalide; 21, 6-shogaol).

Acetyl phenylhydrazine (APH) was obtained from Tianjin Institute of Fine Chemicals retrocession (batch number: 20130816, Tianjin, China). Cyclophosphamide (CP) was purchased from Jiangsu Hengrui Medicine Co., Ltd. (batch number: 15031218). Reference compounds of trigonelline (1), protocatechuic acid (3), hydroxysafflor yellow A (4), amygdalin (6), chaffier acid (7), liquiritin (9), ferulic acid (10), zingerone (11), senkyunolide I (13), senkyunolide H (14), glycyrrhizic acid (16), 6-gingerol (17), ligustilide (19), and butylidenephthalide (20) were purchased from Sichuan Shinning Biotech Institute (Chengdu, China). Stachydrine hydrochloride (2), chlorogenic acid (5), leonurine hydrochloride (8), isoliquiritoside (12), liquiritigenin (15), senkyunolide A (18), 6-shogaol (21) were obtained from National Institute for the Control of Pharmaceutical and Biological Product (Beijing, China). The chemical structures of these chemical standards were given as shown in Figure 1. By using the method of peak area normalization, the purity of all standards was over 98%. Acetonitrile with HPLC grade was obtained from Merck (Darmstadt, Germany); deionized water was purified using a Milli-Q water purification system (Bedford, MA, USA); the remaining reagents were analytical grade.

### 2.2. Herbal Extraction

The mixture (699 g) of DG-YMC-CX-TR-HH-JT-ZGC was mixed (80 : 100 : 30 : 8 : 5 : 5 : 5). Then the mixture was extracted with boiling water (1 : 8, w/v) for three times (2 h, 1.5 h, and 1.5 h). The extract was filtered through gauze, and the filtrate was subsequently evaporated with rotary evaporation under vacuum at 60°C, and the extract of XSHG was finally obtained. To prepare the DY, DC, DT, DH, DJ, and DZ extracts, the mixtures of decomposed recipes were extracted using the same preparation methods. Taking the case of the preparation of sample DY, a total of 159 g mixed powder of CX-TR-HH-JT-ZGC (30 : 8 : 5 : 5 : 5) was decocted by refluxing with water (1 : 8, w/v) for 2 h, 1.5 h, and 1.5 h, respectively. Then the extract was filtered and concentrated to obtain the DY sample. The specific compositions for the different preparations were listed in Table S2.

### 2.3. Evaluation of Hematinic Effects

#### 2.3.1. Animal Treatment

Kunming mice of clean grade (20–25 g) were provided by Shanghai Slac Laboratory Animal Co., Ltd. (Shanghai). And they were kept in an environmentally controlled breeding room (temperature: 20 ± 2°C, humidity: 50 ± 5%) for 10 days before the experiments began. The protocol was carried out in accordance with the Guide for the Care and Use of Laboratory Animals (US National Research Council, 1996), and the Animal Experimental Ethical Committee of Nanjing University of Chinese Medicine. All efforts were made to relief the suffering of animals.

After 10 days of acclimatization, 100 mice were randomly divided into 10 groups with 10 mice in each: the control (K), model (M), positive drug control group (Fu-Fang-A-Jiao-Jiang, AJ), and the groups XSHG, DY, DC, DT, DH, DJ, and DZ. Except the control group, the mice in other groups were hypodermically injected with 2% APH saline solution at dose of 20 mg·kg^−1^ and 10 mg·kg^−1^ body weight on days 1 and 4 accordingly; 2 h after the hypodermic injection with APH saline solution on day 4, the mice were injected with CP saline solution intraperitoneally at a dose of 20 mg·kg^−1^ on days 4, 5, 6, and 7 [16]. As a result, HAA mice were induced, and the experimental period was 7 days.

The mice in positive drug control group were orally given Fu-Fang-A-Jiao-Jiang at a dose of 0.1 g·kg^−1^, and the mice in XHSG group were intragastrically administrated with XHSG extracts at a dose of 4.86 g·kg^−1^. By using the body surface area normalization method, the animal dose of XSHG, DY, DC, DT, DH, DJ, and DZ groups was inferred based on the human daily dose. And the formula was described as follows: human dose of crude herbs in clinic × 0.018/200 × 1000 × the multiple of clinical equivalency dose [8]. The dose of XSHG was equivalent to one time of the adult daily dose of the formula XSHG crude drugs (36 g, from XSHG in which DG-YMC-CX-TR-HH-JT-ZGC were 12.36 g, 15.45 g, 4.64 g, 1.24 g, 0.77 g, 0.77 g, and 0.77 g, respectively) according to clinical prescription. And the doses of DY, DC, DT, DH, DJ, and DZ extracts were 1.11, 2.57, 3.02, 3.09, 3.09, and 3.09 g·kg^−1^, respectively. Control and model groups were orally given the same volume of saline solution. All animals were intragastrically administrated one time each day for continuous 7 days since the beginning of reproducing HAA model.

#### 2.3.2. Sample Collection

Before the gavage administration on day 7, the mice of all groups were weighted by electronic balance. Half hour after the last administration of the extracts, blood samples (0.5 mL) were collected by orbital venous plexus method into 1.5 mL centrifuge tube with 1.5% EDTA-2Na (EDTA-2Na/blood: 1/9, v/v). Then blood samples were used to measure peripheral blood routine and ATPase activities of red cell membrane. Moreover, the whole liver, spleen, and thymus of mice in every group were isolated and then weighed by electronic balance. All experiments were finished within 3 h after sample collection.

#### 2.3.3. Effect Indicators of Nourishing Blood

A total of 300 *μ*L blood was applied to measure the peripheral blood routine by Sysmex XS 800i (Shanghai, China) type fully automatic blood analyzer. And the peripheral blood routine in this experiment includes white blood cell count (WBC/10^9^ L^−1^), red blood cell count (RBC/10^12^ L^−1^), hemoglobin (HGB/g·L^−1^), neutrophils ratio (NE/%), lymphocyte ratio (LY/%), monocyte ratio (MO/%), haematocrit (HCT), and platelet count (PLT/10^9^ L^−1^). Also, thymus index, spleen index, and liver index of mice in every group were obtained according to the following equation: thymus index = the weight of thymus/the body weight of mice; spleen index = the weight of spleen/the body weight of mice; liver index = the weight of liver/the body weight of mice, respectively.

Additionally, the ATPase activities of red cell membrane, including Na^+^-K^+^ and Ca^2+^-Mg^2+^ ATPase, were carried out in a 96-well microplate reader as reported before. By using commercial Na^+^, K^+^ and Ca^2+^, Mg^2+^ ATPase kits (Nanjing Jiancheng Bioengineering Institute, China), the ATPase activities were assayed by measuring the release of inorganic phosphate (Pi) from the hydrolysis of ATPase according to the manufacturer's instructions. Briefly, the activities of Na^+^-K^+^ and Ca^2+^-Mg^2+^ ATPase were determined by measuring the amount of inorganic phosphate with malachite green dye method and then expressed as micromoles per milliliter blood. By using a malachite-based Biomol Green reagent, released inorganic phosphate (Pi) was assayed [17].

### 2.4. Quantitative Analysis of Marker Components

#### 2.4.1. Preparation of Standard Solutions

A mixed stock solution containing the reference standards 1–21 was prepared by dissolving them in 90% methanol and then this mixed standard stock solution was diluted with 90% methanol to a series of appropriate concentrations for the construction of calibration curves. All the samples were stored at 4°C until use and filtered through a 0.22 *μ*m cellulose membrane prior to injection.

#### 2.4.2. Preparation of Sample Solutions

The concentrated extracts of XSHG, DY, DC, DT, DH, DJ, and DZ for the treatment of mice were diluted to 100 times with Millipore water, respectively. Subsequently, the extracts of different samples were centrifuged at 13000 rpm for 10 min, and the supernatant was stored at 4°C. Before the UHPLC-TQ-MS/MS analysis, the sample solutions were filtered through 0.22 *μ*m membrane.

#### 2.4.3. UHPLC-TQ-MS/MS Instrumentation and Conditions

A Waters ACQUITY UHPLC system (Waters, Milford, MA, USA) was performed comprised of a binary pump solvent delivery system, an online degasser, and an autosampler. Chromatographic separations were carried out on a Thermo Scientific Hypersil GOLD (100 mm × 3 mm, 1.9 *μ*m) column. The mobile phase was composed of A (acetonitrile) and B (water with 0.1% formic acid) using a gradient program as follows: 0–2 min, isocratic 5% A; 2–12 min, linear gradient 5%–40% A; 12–18 min, linear gradient 40%–95% A; 18-19 min, linear gradient 95%–5% A; and 19-20 min, isocratic 5%–5% A. The flow rate of the mobile phase was set at 0.40 mL·min^−1^, and the injection volume was 2 *μ*L. The column temperature and the autosampler were conditioned at 35°C and 4°C, respectively.

Mass spectrometry analysis was operated using a Xevo Triple Quadrupole tandem quadrupole mass spectrometer (Waters Corp., Milford, MA, USA) equipped with an electrospray ionization source (ESI). The mass data was collected from *m*/*z* 100 to 1000, and the TQ mass spectrometer was performed in both positive and negative modes. Additionally, multiple reaction monitoring (MRM) was applied for the MS spectra. The parameters of MS analysis were as follows: drying gas, N_2_; cone gas flow, 50 L·h^−1^; desolvation gas flow, 1000 L·h^−1^; capillary voltage, 3000 V; desolvation temperature, 550°C; source temperature, 150°C. Dwell time was automatically designed by software. The collision energy and cone voltage were optimized for each compound, and the selected values are shown in Table S3.

#### 2.4.4. UHPLC-TQ-MS/MS Method Validation

The UHPLC method was validated for linearity, limits of quantification and detection (LOQs and LODs), interday and intraday precision, stability, accuracy, and matrix effect according to some reports on quantification analysis.

A series of standard solutions containing the 21 analytes were injected in duplicate, and the calibration curves could be constructed by the peak areas versus the corresponding concentrations of the analytes. The values of LOD and LOQ were acquired using diluted standard solution when the signal-to-noise ratios (S/N) were about 3 and 10, respectively.

To evaluate the precision of the developed method, six replicates of the standard solution were examined for intraday precision, and then the sample was further analyzed during consecutive three days for interday precision. To estimate the repeatability, six independent sample solutions (XSHG sample) were prepared by the above method, and the variations of these samples were determined by UHPLC-TQ-MS/MS. One of XSHG sample solutions was kept at room temperature and analyzed at 0, 2, 4, 8, 12, 18, and 24 h, respectively, to evaluate the stability of these samples.

The accuracy of this method was confirmed with the spike recovery test. The recovery was performed by spiking the marker analytes with low (50%), middle (100%), and high (150%) levels to 1.0 g of the XSHG sample, which had been analyzed in the previous study. The triplicate spiked samples were then extracted and processed according to the sample preparation methods mentioned above. To compare the spiked samples, a blank sample without standards was also similarly prepared and analyzed.

Because the ion enhancement or suppression could have the negative impact on the analysis of these compounds, the slope comparison method was applied to study the matrix effect [18]. For the construction of standard addition calibration curves, the sample extracts were spiked with appropriate amounts of standards to perform the recovery measurement. Subsequently, the slopes of calibration curves from standard addition experiments were compared with the slopes of calibration curves from 90% methanol standards under the same concentration levels; thus the ratio (slope matrix/slope solvent) was used to evaluate the matrix effect.

### 2.5. Statistical Analyses

All of the results were presented as mean ± standard deviation (SD) and the statistical program SPSS 19.0 was applied to analyze their variations. Additionally, one-way analysis of variance (ANOVA) with Dunnett's test was used to calculate the statistical comparison. In all cases, *P* < 0.05 was regarded to be significant.

## 3. Results

### 3.1. Evaluation of Hematinic Function

#### 3.1.1. Peripheral Blood Routine Analysis

The peripheral blood routine data are shown in Table 1. Compared with control group, all the indicators of peripheral blood routine of model group had statistical differences (*P* < 0.05). Among them, HGB, RBC, LY%, and HCT in model group were obviously decreased in comparison with control group (*P* < 0.01), while the indicators of WBC, NE%, MO%, and PLT increased significantly (*P* < 0.01). All of the results above demonstrated that the blood deficiency mice were successfully induced. Compared with model group, WBC, MO%, and HCT% in XHSG group and the groups of decomposed recipes were significantly reduced (*P* < 0.05); except for the DY and DC group, RBC, HGB, and LY (%) in other groups were obviously increased (*P* < 0.05); NE (%) and PLT in the DT group decreased significantly (*P* < 0.05). And in comparison with the XSHG group, except WBC, NE (%), and MO (%), other indicators in DY group had remarkable difference (*P* < 0.01); RBC, HGB, LY (%), and PLT in DC group showed statistical difference (*P* < 0.05); WBC and LY (%) in DH group changed significantly (*P* < 0.05); LY (%) in the DT, DJ, and DZ groups were remarkably reduced (*P* < 0.05).

#### 3.1.2. Effects on Immune Organ Index

**Figure 2:**
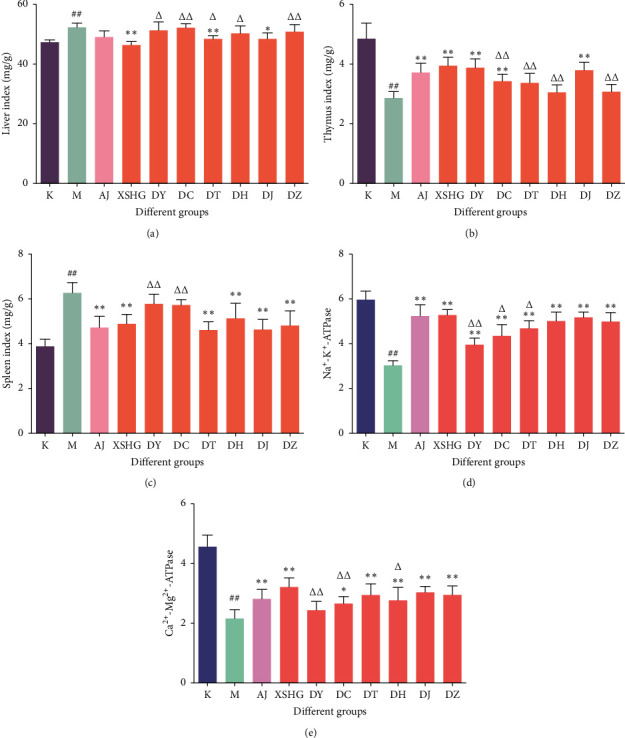
The effects of immune organ index and ATPase activities for administration groups in HAA mice ($\bar{x}\pm s$, *n* = 10). (a) Liver index; (b) thymus index; (c) spleen index; (d) Na^+^-K^+^-ATPase; (e) Ca^2+^-Mg^2+^-ATPase). ^#^*P* < 0.05 and ^##^*P* < 0.01 versus control group; ^*∗*^*P* < 0.05 and ^*∗∗*^*P* < 0.01 versus model group; ^Δ^*P* < 0.05 and ^Δ^*P* < 0.01 versus XSHG group. K, control group; M, model group; XSHG, Xin-Sheng-Hua granules; DY, XSHG minus Danggui-Yimucao; DC, minus Danggui-Chuanxiong; DT, minus Danggui-Taoren; DH, minus Danggui-Honghua; DJ, minus Danggui-Jiangtan; DZ, minus Danggui-Zhigancao.

The effects on immune organ index for each group are shown in Figure 2. As shown in Figures 2(a)–2(c), in the model group, thymus index was decreased, while liver index and spleen index increased conspicuously (*P* < 0.01). In the XSHG and DJ groups, the liver index and spleen index were significantly reduced and the thymus index was increased obviously (*P* < 0.05). Thymus index in DY and DC groups had statistical difference (*P* < 0.01), and the DT group could significantly affect the liver index and spleen index (*P* < 0.01) compared to the model group. Moreover, the DH and DZ groups had significant effects on the spleen index (*P* < 0.01). Compared with the XSHG group, except DJ group, liver index in other groups significantly increased (*P* < 0.05); thymus index in DC, DT, DH, and DZ groups obviously reduced (*P* < 0.05); and spleen index in DY and DC groups was conspicuously increased (*P* < 0.01).

#### 3.1.3. Effects on ATPase Activity

As shown in Figures 2(d) and 2(e), the Na^+^-K^+^ and Ca^2+^-Mg^2+^ ATPase activities of red cell membrane for the model group were significantly reduced compared to the control group (*P* < 0.01), indicating that the blood deficiency mice could affect the ATPase activities. After administration, all groups could obviously improve the Na^+^-K^+^ ATPase activity (*P* < 0.01); except the DY group, the Ca^2+^-Mg^2+^ ATPase activities in other groups could be significantly increased (*P* < 0.05). Compared to the XSHG group, the Na^+^-K^+^ and Ca^2+^-Mg^2+^ ATPase activities of red cell membrane for DY and DC groups were reduced conspicuously (*P* < 0.05); and the Na^+^-K^+^ ATPase activity in DT group and the Ca^2+^-Mg^2+^ ATPase activity for DH group significantly decreased (*P* < 0.05).

### 3.2. Integrated Effects of Hematinic Function

**Figure 3:**
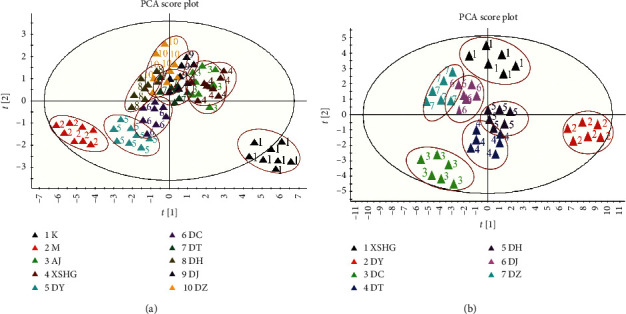
PCA score plot between control group and dose groups (Model, XSHG, DY, DC, DT, DH, DJ, and DZ). (a) 2D plot of the integrated hematinic effects; (b) 2D plot of the amounts of the 21 analytes in different samples.

To investigate the integrated effects of hematinic function, the raw data of effect indicators in different groups were imported into EZinfo 2.0 software for PCA analysis. In the PCA scores (Figure 3(a)), each point represented an individual sample and the similar effects of the administration groups would be clustered together. Clear separation between control and model group from the PCA scores could be easily seen, and other treatment groups scattered across these two groups. These results suggested that the model and control group had different effect indicators, indicating that the blood deficiency mice were successfully copied.

Furthermore, the relative distances between administration groups (the model, XSHG, DY, DC, DT, DH, DJ, and DZ groups) and the control group from PCA score plot of effect indicators were calculated for quantization [10, 19]. The jumping-off point of the integrated effects for treatment groups was defined as the mean integration effects for normal rats from PCA; then the relative distances of different drug groups were obtained. From Table S4, it was found that the relative distances of the treatment groups were decreased compared to model group, suggesting that the samples of XHSG and its decomposed recipes could regulate the HAA mice to the normal state. Remarkably, the distances of DY and DC groups were relatively long, while the relative distances of DJ and DT groups were shorter than the others, which indicated that the DY and DC groups had poor curative effects on the treatment of anemia mice. And the orders of hematinic effects of dose groups were as follows: XHSG > DJ > DT > DZ > DH > DC > DY. These results demonstrated that the herb pairs containing Danggui played different roles in this formulae, and herb pairs Danggui-Yimucao and Danggui-Chuanxiong made great contribution to hematinic effects of XSHG.

### 3.3. Quantitative Analysis of Samples Using UHPLC-TQ-MS/MS

#### 3.3.1. Optimization of the Chromatographic Conditions and Mass Spectrometric Conditions

**Figure 4:**
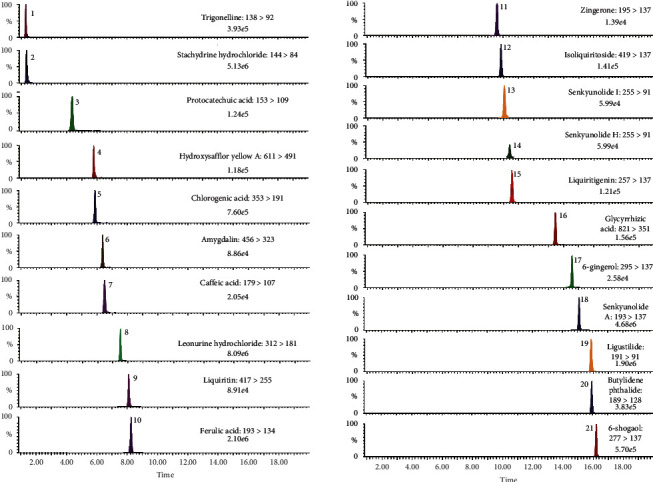
The UHPLC-TQ-MS/MS analysis MRM chromatogram of 21 analytes in the samples of XSHG and its decomposed recipes.

Due to various structural types of active components in the sample XSHG, the UHPLC conditions were studied on a Thermo Scientific Hypersil GOLD column (100 mm × 3 mm, 1.9 *μ*m). Compared with methanol, better separation efficiency could be achieved for the low polarity components (gingerols) when using acetonitrile. And the additives of formic acid could reduce the peak tailing and increase the retention time of aromatic acids. Finally, the mobile phase of 0.1% formic acid solution, acetonitrile, was selected. For the good separation of high polarity constituents (alkaloids), the initial mobile phase was adjusted at 95% aqueous phase—5% organic phase. Although stachydrine hydrochloride and trigonelline had similar retention time on this chromatographic condition, the two components could be distinguished by their characteristic fragmentation. The flow rate and column temperature were set at 0.4 mL·min^−1^ and 40°C. Typical chromatograms of multiple components with MRM mode are shown in Figure 4.

The target compounds were examined separately under the ESI^+^ or ESI^−^ mode according to the intensity and sensitivity of their signals. To obtain the characteristic transition for MRM determination of each compound, at least two precursor/product ion pairs of the compound were chosen in this investigation. By comparing the response of the obtained ion pairs, the highest sensitive and specific ion pairs were finally applied for MS/MS detection. Afterwards, the values of collision energy and cone voltage were optimized by the Intellistart program. In this study, protonated molecules [M + H]^+^ were determined with abundant ions in the analysis of phthalides, alkaloids, and gingerols, while organic acids, flavonoids, and amygdalin exhibited deprotonated molecules [M – H]^−^ with the most abundant intensities. For the gingerols (6-gingerol, 6-shogaol, and zingerone), *m*/*z* 137 as the daughter ions could be observed in these analytes. Additionally, *m*/*z* 137 could also be detected in the MS fragment ions of senkyunolide A and liquiritigenin. As for senkyunolide I and senkyunolide H, they could produce the same precursor/product ion pairs of 225 ⟶ 91, thus leading to the mutual interference. Fortunately, the two analytes could be baseline separated under the optimized UHPLC conditions. All the MRM transitions and parameters optimized in the investigation are summarized in Table S3.

#### 3.3.2. Method Validation

As shown in Table S5, the correlation coefficient values greater than 0.9937 were obtained for all the standards within the test ranges. Their values of LOD and LOQ for MS/MS determination were in the ranges from 0.62 to 9.89 ng/mL and 1.96 to 30.58 ng/mL, respectively. Other results about method validation are listed in Table S6. The overall intra- and interday variations (RSDs) of the 21 standards ranged from 1.06% to 3.26% and 1.43% to 4.38%, respectively. The repeatability and stability expressed as RSDs were in the ranges of 1.85%–4.86% and 1.67%–4.59%, respectively. The recoveries of 21 analytes ranged from 94.79% to 102.76% with RSD between 1.31% and 4.78%. Additionally, the slop ratio values of matrix curve to the neat standard solution curve ranged from 0.91 to 1.07, suggesting that the matrix effect was not significant under the optimized conditions. Consequently, these results revealed that the proposed UHPLC-TQ-MS/MS method was accurate, sensitive, and repeatable for the quantitation of these target constituents.

#### 3.3.3. Quantitative Analysis of Samples

Different samples of XSHG and the disassembled recipes, including DY, DC, DT, DH, DJ, DZ, and XSHG, were subsequently determined by the validated UHPLC-TQ-MS/MS method. And the observed amounts of the individual analyte in these samples are presented in Table 2, respectively. In order to study the roles of herb pairs containing Danggui on the formulae, the theorized amounts of the analytes in disassembled prescription were originally introduced. Apart from aromatic acids and phthalides, other analytes were the marker compounds and each compound could only be found in one certain plant; thus, the theorized amounts of these analytes in discomposed recipes were their observed amounts in XSHG. For example, stachydrine hydrochloride could only be found in herb Yimucao, and the amount of stachydrine hydrochloride in XSHG was 2148.92 mg, and then the theorized amount of stachydrine hydrochloride in the decomposed recipes was 2148.92 mg.

Annoyingly, aromatic acids and phthalides could be detected in Danggui and Chuanxiong, while these constituents could just be found from Chuanxiong in the disassembled samples. To solve the problem, aromatic acids and phthalides were also determined in the single herbs of Danggui (240 g) and Chuanxiong (90 g), and the preparation of these samples all adopted the above extraction methods. The results (Table S7) showed that the amounts of these target compounds were different in Danggui and Chuanxiong. Then the proportionality coefficient of aromatic acids or phthalides in Chuanxiong could be obtained according to the following equation: (Amount in CX (90 g))/(Amount in CX (90 g) + Amount in DG (240 g)). For example, the amount of ligustilide in the single herbs of Chuanxiong (90 g) and Danggui (240 g) was 242.28 mg and 453.36 mg, respectively; thus the proportionality coefficient of ligustilide in Chuanxiong could be calculated: 242.28 mg/(242.28 mg + 453.36 mg) = 0.348. Afterwards, the theorized amounts of these constituents in XSHG (only from Chuanxiong) could be further deduced: Amount_The._ = Proportionality coefficient of CX × Amount of the analyte in XSHG. Using ligustilide as a case, the proportionality coefficient of ligustilide in CX was 0.348 according to the above analysis result, and the amount of ligustilide in XSHG 572.22 mg; then the theorized amount of ligustilide could be calculated: Amount_The._ = 0.348 × 572.22 mg = 199.26 mg.

The theorized amounts of the compounds in XSHG are also presented in Table 2. Although there were some deficiencies in the calculation of theorized amounts for aromatic acids and phthalides, the proportionality coefficient of these constituents in Danggui and Chuanxiong might be an effective way to calculate the theorized amounts of Chuanxiong in the disassembled formula.

### 3.4. The Concentration Variations of Target Compounds and Chemical Interaction Analysis

To discover the potential reasons of the contribution of herb pairs containing Danggui to XSHG, the source data was firstly standardized by the statistical program SPSS 19.0; then standardized data was imported into MassLynx software [19, 20]. And the PCA score plot (two principal components) of seven different groups is given in Figure 3(b). Compared to other samples, the sample DJ and DZ were closer to XSHG, while the sample DY and DC were farther away from this formula. These results indicated that each herb pair was the indispensable part of the formulae, and herb pairs Danggui-Yimucao, Danggui-Chuanxiong might be particularly important for this formula.

**Figure 5:**
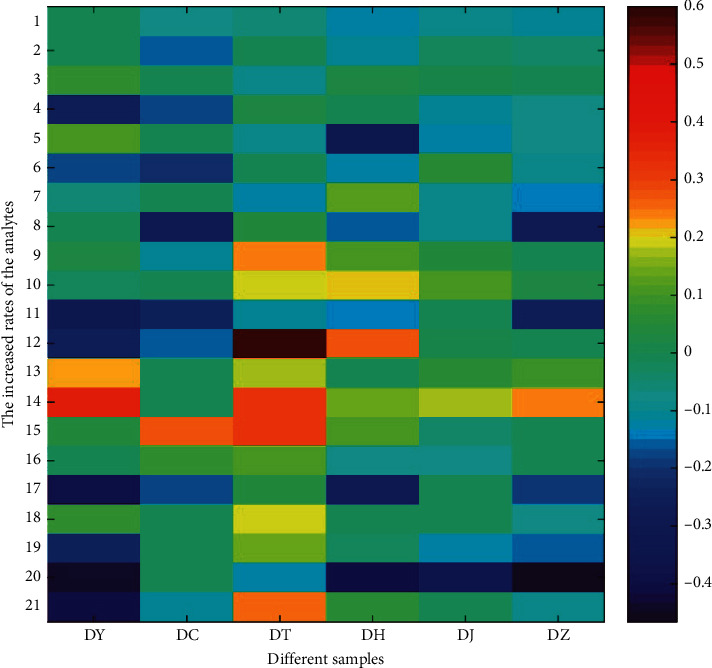
The heat map of increased rates of target constituents in six different separated prescriptions (DY, DC, DT, DH, DJ, and DZ). The increased rate of 0 indicates that the observed amount was equal to its theorized amount, otherwise denoting amount increases (>0) or amount decreases (<0) in the separated prescriptions.

Moreover, the increased rate = (Amount_Obs._ − Amount_The._)/Amount_The_. was applied to evaluate the amount variations of different analytes in decomposed recipes. Based on the above equation, the increased rates of active constituents were calculated accordingly. And the heat map of the increased rates of target constituents in different separated prescriptions is given in Figure 5. In the heat map, the increased rate of 0 indicates that the observed amount was equal to its theorized amount, otherwise denoting amount increases (>0) or amount decreases (<0). Altogether, the increased rates of active constituents in the sample DJ and DT were higher than those in other samples, while the increased rates of target compounds in the sample DC and DY were lower than those in other samples. The heat map illustrated that herb pairs containing Danggui played significant roles in XSHG, and various herb pairs might make different contribution to the formula.

These results above suggested that vast majority of active compounds decreased to some extent when herb pairs Danggui-Yimucao and Danggui-Chuanxiong were eliminated from XSHG. Thus, this fact could be the importantly potential reason of different contributions of herb pairs containing Danggui to XSHG on HAA mice.

## 4. Discussion

TCM formulae were the indispensable part of human life, which play significant roles for disease treatment in the developing countries. However, the obscure composition of TCM medicines limited their clinical application [21]. Lots of efforts have been devoted to clarifying the effective material basis of TCM medicines, such as isolating and identifying single effective ingredients [22–24]. However, these studies disregarded the integrative therapeutic effects of multiple effective compounds in TCM medicines.

Herb pairs are the basis units of TCM formulae, which are formed by two herbs. The study of herb pairs could help to discover the effective material basis of the relevant TCM formulae [25]. XSHG prescription was composed of seven herbs, including Danggui, Yimucao, Chuangxiong, Taoren, Honghua, Jiangtan, and Zhigancao. In the clinic, XSHG could exhibit good therapeutic effects on anemia via increasing hematopoietic stem cell differentiation and proliferation [9]. Strikingly, different herb pairs could be formed when the herb Danggui was combined with other six herbs [6]. Therefore, the herb pairs containing Danggui could play significant roles for its treatment of anemia. To investigate the potential effective material basis, the roles of herb pairs containing Danggui in XSHG were comparatively evaluated using withdraw analysis. Fu-Fang-A-Jiao-Jiang is a famous TCM formula descending from “*Jing Yue Quan Shu*” (Ming Dynasty). It has been approved by National Medical and Products Administration (NMPA) to replenish qi and nourish blood. Modern research revealed that Fu-Fang-A-Jiao-Jiang could effectively promote hematopoietic effects through improving bone marrow hematopoietic microenvironment; thus, it was selected as positive drug in this study [26].

Combining the chemical analysis and bioactive evaluation, the results discovered that herb pairs Danggui-Yimucao and Danggui-Chuanxiong made great contributions to the treatment effects of XSHG. Danggui, which is also called “female ginseng,” has good effects for the treatment of complicated gynecological disorders through nourishing and tonifying blood [27]. Yimucao has the effects on promoting blood flow for regulating menstruation, and it has been extensively used to treat postpartum hemorrhage [28]. Chuanxiong could exert key roles in regulating menstruation and relieving pain and activating blood circulation [29]. Modern research has demonstrated that Danggui-Chuanxiong showed obvious synergistic effects on regulating peripheral blood routine, energy metabolic enzymes, and immune organs [30, 31]. In our study, when the herb pair Danggui-Chuanxiong was excluded from XSHG, the hematinic function decreased obviously, which was consistent with the previous studies [30, 31].

## 5. Conclusions

The withdrawal analysis method was applied to evaluate the contribution of herb pairs containing Danggui to XSHG on HAA mice. With PCA analysis, a clear separation of healthy control group and deficiency group was obtained, and the administration groups were located between model group and control group. Among these dose groups, the group XSHG was the closest to the control group, while the group DY was the farthest away from the control group. In order to reveal the potential reasons of different contributions of six herb pairs to XSHG, the content variations of these samples were also analyzed by UHPLC-TQ-MS/MS method. And the contents of target constituents in XSHG underwent different changes when herb pairs containing Danggui were excluded from this formula, respectively. In particular, the contents variations of DY and DC groups mostly decreased to some extent compared with that of XSHG group. The data of pharmacodynamics and the contents changes of the analytes in the decomposed recipes suggested similar results that a series of herb pairs containing Danggui were indispensable parts of the formulae, and herb pairs Danggui-Yimucao might be particularly important. Our results in the present study could lay foundation to further reveal the compatibility mechanism of XSHG. Furthermore, this original method might provide a good reference for interpreting the drug interactions from the aspects of herb pairs.

## Figures and Tables

**Table 1:** The results of peripheral blood routine between control group and treatment groups ($\bar{x}+s$, *n* = 8).

Group	WBC (10^9^/L)	RBC (10^12^/L)	HGB (g/L)	NE (%)	LY (%)	MO (%)	HCT (%)	PLT (10^9^/L)
Control	5.08 ± 0.82	8.04 ± 0.73	144.88 ± 5.33	15.05 ± 1.51	71.15 ± 3.34	17.49 ± 2.23	39.35 ± 2.04	366.00 ± 19.21
Model	9.11 ± 1.18^##^	6.31 ± 0.32^##^	128.88 ± 6.31^##^	30.36 ± 2.30^##^	55.23 ± 4.38^##^	24.15 ± 2.19^##^	28.91 ± 2.18^##^	520.38 ± 49.41^##^
AJ	5.26 ± 0.84^*∗∗*^	7.58 ± 0.47^*∗∗*^	141.13 ± 5.08^*∗∗*^	28.46 ± 2.69	64.91 ± 3.24^*∗∗*^	18.61 ± 1.32^*∗∗*^	38.01 ± 2.13^*∗∗*^	447.25 ± 28.97^*∗∗*^
XSHG	5.73 ± 0.96^*∗∗*^	7.66 ± 0.32^*∗∗*^	140.75 ± 5.18^*∗∗*^	28.89 ± 1.54	66.45 ± 3.06^*∗∗*^	19.49 ± 1.11^*∗∗*^	37.03 ± 2.24^*∗∗*^	481.75 ± 40.75
DY	6.49 ± 0.82^*∗∗*^	6.53 ± 0.37^∆∆^	130.50 ± 4.24^∆∆^	27.68 ± 3.21	57.40 ± 3.87^∆∆^	21.21 ± 2.36^*∗*^	33.05 ± 3.23^*∗∗*^^∆∆^	542.13 ± 47.43^∆∆^
DC	6.28 ± 0.58^*∗∗*^	6.81 ± 0.52^∆∆^	131.25 ± 5.90^∆∆^	28.65 ± 1.97	59.33 ± 2.96^∆∆^	21.09 ± 1.72^*∗*^	34.56 ± 1.54^*∗∗*^	529.88 ± 22.96^∆^
DT	6.58 ± 1.19^*∗∗*^	7.40 ± 0.54^*∗∗*^	139.13 ± 3.80^*∗∗*^	26.40 ± 3.06^*∗∗*^	61.06 ± 3.17^*∗∗*^^∆^	20.66 ± 2.16^*∗∗*^	37.74 ± 1.95^*∗∗*^	469.63 ± 28.39^*∗*^
DH	7.08 ± 0.60^*∗∗*^^∆^	7.20 ± 0.41^*∗∗*^	136.38 ± 3.66^*∗*^	28.84 ± 2.13	60.16 ± 4.08^*∗*^^∆∆^	21.18 ± 2.23^*∗*^	36.03 ± 1.87^*∗∗*^	512.75 ± 46.41
DJ	6.44 ± 0.99^*∗∗*^	7.56 ± 0.39^*∗∗*^	143.38 ± 4.75^*∗∗*^	28.76 ± 2.03	62.05 ± 2.87^*∗∗*^^∆^	20.74 ± 1.65^*∗∗*^	36.21 ± 2.64^*∗∗*^	487.38 ± 29.18
DZ	6.78 ± 1.04^*∗∗*^	7.46 ± 0.34^*∗∗*^	138.88 ± 5.03^*∗∗*^	30.09 ± 2.46	60.13 ± 2.98^*∗*^^∆∆^	20.96 ± 1.52^*∗∗*^	35.61 ± 1.63^*∗∗*^	489.00 ± 27.92

^#^ *P* < 0.05 and ^##^*P* < 0.01 versus control group; ^*∗*^*P* < 0.05 and ^*∗∗*^*P* < 0.01 versus model group; ^Δ^*P* < 0.05 and ^ΔΔ^*P* < 0.01 versus XSHG group. WBC: white blood cell count; RBC: red blood cell count; HGB: hemoglobin; NE: neutrophil ratio; LY: lymphocyte ratio; MO: monocyte ratio; HCT: haematocrit; PLT: platelet count.

**Table 2:** Amounts (mg) of the 21 compounds in XSHG and six samples of decomposed recipes (*n* = 6).

Analyte	Samples (mg, *n* = 6)							
	XSHG_Obs._^a^	Sam_The._^b^	DY_Obs._^a^	DC_Obs._^a^	DT_Obs._^a^	DH_Obs._^a^	DJ_Obs._^a^	DZ_Obs._^a^
1	208.01 ± 8.45	208.01 ± 8.45	n.d.	191.10 ± 6.87^*∗∗*^	196.70 ± 4.52^*∗*^	182.30 ± 8.73^*∗∗*^	187.95 ± 4.32^*∗∗*^	183.92 ± 7.95^*∗∗*^
2	2148.92 ± 103.41	2148.92 ± 103.41	n.d.	1820.32 ± 67.63^*∗∗*^	2130.15 ± 91.83	1924.15 ± 48.82^*∗∗*^	2087.40 ± 84.16	2050.64 ± 84.67^*∗*^
3	38.78 ± 4.30	19.53 ± 1.02	21.08 ± 1.71^#^	n.d.	17.66 ± 1.26^*∗∗*^	19.95 ± 1.46	19.70 ± 1.11	19.32 ± 1.20
4	507.14 ± 27.22	507.14 ± 27.22	374.82 ± 24.23^*∗∗*^	419.03 ± 14.53^*∗∗*^	517.11 ± 19.44	n.d.	453.51 ± 23.24^*∗∗*^	467.63 ± 22.91^*∗*^
5	100.93 ± 5.07	56.16 ± 3.06	62.07 ± 2.14^##^	n.d.	50.73 ± 2.73^*∗∗*^	39.30 ± 2.14^*∗∗*^	48.78 ± 2.42^*∗∗*^	51.68 ± 1.73^*∗∗*^
6	430.58 ± 36.53	430.58 ± 36.53	358.24 ± 23.02^*∗∗*^	342.14 ± 18.58^*∗∗*^	n.d.	377.47 ± 15.62^*∗∗*^	456.07 ± 26.64^##^	390.51 ± 15.81^*∗∗*^
7	54.34 ± 3.60	25.56 ± 1.39	24.18 ± 1.51	n.d.	22.49 ± 1.35^*∗∗*^	28.62 ± 1.47^##^	23.31 ± 1.34^*∗*^	21.84 ± 1.17^*∗∗*^
8	95.45 ± 4.03	95.45 ± 4.03	n.d.	66.95 ± 4.10^*∗∗*^	99.45 ± 6.25	80.20 ± 4.03^*∗∗*^	86.72 ± 5.39^*∗∗*^	69.60 ± 3.66^*∗∗*^
9	73.55 ± 3.35	73.55 ± 3.35	75.77 ± 4.06	65.99 ± 2.94^*∗∗*^	91.00 ± 2.79^##^	81.04 ± 2.68^##^	76.15 ± 2.19	n.d.
10	267.52 ± 20.76	82.98 ± 5.75	74.16 ± 3.52	n.d.	99.20 ± 8.67^##^	99.87 ± 7.65^##^	91.91 ± 7.95^##^	85.35 ± 6.04
11	21.93 ± 1.43	21.93 ± 1.43	15.27 ± 1.31^*∗∗*^	16.51 ± 1.58^*∗∗*^	19.44 ± 1.24^*∗*^	18.68 ± 1.40^*∗∗*^	n.d.	16.45 ± 1.07^*∗∗*^
12	2.17 ± 0.17	2.17 ± 0.17	1.62 ± 0.11^*∗∗*^	1.82 ± 0.12^*∗∗*^	3.48 ± 0.22^##^	2.77 ± 0.12^##^	2.20 ± 0.16	n.d.
13	134.92 ± 7.07	60.39 ± 2.26	74.46 ± 2.47^##^	n.d.	70.98 ± 3.34^##^	59.66 ± 1.82	63.81 ± 1.57	65.80 ± 1.81^#^
14	73.81 ± 6.36	26.46 ± 1.79	36.44 ± 2.55^##^	n.d.	34.83 ± 1.05^##^	30.17 ± 1.67^##^	30.87 ± 0.97^##^	32.57 ± 1.72^##^
15	2.51 ± 0.24	2.51 ± 0.24	2.62 ± 0.22	3.18 ± 0.14^##^	3.30 ± 0.24^##^	2.75 ± 0.22	2.41 ± 0.16	n.d.
16	430.17 ± 20.84	430.17 ± 20.84	423.49 ± 22.98	460.62 ± 21.44^##^	474.48 ± 28.63^##^	395.46 ± 15.28^*∗∗*^	397.73 ± 19.17^*∗*^	n.d.
17	47.87 ± 2.69	47.87 ± 2.69	28.97 ± 2.00^*∗∗*^	39.33 ± 2.60^*∗∗*^	49.91 ± 3.02	33.66 ± 2.62^*∗∗*^	n.d.	39.09 ± 2.24^*∗∗*^
18	437.86 ± 32.07	173.97 ± 10.72	187.92 ± 17.68	n.d.	206.68 ± 12.24^##^	172.80 ± 7.15	172.44 ± 11.35	162.31 ± 11.21^*∗*^
19	572.17 ± 33.48	199.26 ± 9.15	152.60 ± 6.28^*∗∗*^	n.d.	228.98 ± 14.30^##^	195.75 ± 13.48	175.37 ± 6.65^*∗*^	166.62 ± 7.41^*∗∗*^
20	36.96 ± 3.61	20.07 ± 0.56	11.25 ± 0.62^*∗∗*^	n.d.	17.47 ± 0.88^*∗∗*^	11.79 ± 0.63^*∗∗*^	12.77 ± 0.75^*∗∗*^	10.68 ± 0.79^*∗∗*^
21	1.43 ± 0.10	1.43 ± 0.10	0.84 ± 0.059^*∗∗*^	1.26 ± 0.09	1.78 ± 0.12^##^	1.52 ± 0.12	n.d.	1.31 ± 0.08^*∗∗*^

n.d. = not detectable; ^*∗*^*P* < 0.05 and ^*∗∗*^*P* < 0.01 (the observed amount decreased in comparison with its theory amount); ^#^*P* < 0.05 and ^##^*P* < 0.01 (the observed amount increased in comparison with its theory amount). ^a^Obs., the observed amount of each compound in different samples. ^b^The theorized amount of each compound in the samples of decomposed recipes. Except for aromatic acids and phthalides, the theorized amounts of other compounds in the decomposed recipes were the observed amounts of the analytes in XSHG accordingly; for aromatic acids and phthalides, the theorized content could be calculated: Amount_The._ = Proportionality coefficient of CX × Amount of the analyte in XSHG. 1, trigonelline; 2, stachydrine hydrochloride; 3, protocatechuic acid; 4, hydroxysafflor yellow A; 5, chlorogenic acid; 6, amygdalin; 7, caffeic acid; 8, leonurine hydrochloride; 9, liquiritin; 10, ferulic acid; 11, zingerone; 12, isoliquiritoside; 13, senkyunolide I; 14, senkyunolide H; 15, liquiritigenin; 16, glycyrrhizic acid; 17, 6-gingerol; 18, senkyunolide A; 19, ligustilide; 20, butylidenephthalide; 21, 6-shogaol.

## Data Availability

The data used to support the findings of this study are available from the corresponding author upon request.

## Supplementary Materials

Supplementary Materials Table S1: the information of the tested samples. Table S2: the specific composition for the different preparations. Table S3: precursor/product ion pairs and parameters for MRM of 21 components of XSHG. TABLE S4: relative distance for hematinic function evaluation between dose groups and control group from the PCA score plot of XSHG and decomposed recipes groups. Table S5: calibration curves, correlation coefficients, linearity ranges, and LOD and LOQ of the 21 investigated components. Table S6: precision, repeatability, stability, recovery, and the matrix effects of the 21 investigated compounds. Table S7: amounts (mg) of the aromatic acids and phthalides in the single herbs of Danggui and Chuanxiong.
