# Supplementary material for: Comparatively Evaluating the Role of Herb Pairs Containing Angelicae Sinensis Radix in Xin-Sheng-Hua Granule by Withdrawal Analysis
Source: Evid Based Complement Alternat Med. 2020 Sep 22;2020:9456350. doi: 10.1155/2020/9456350 (PMC7528019; doi:10.1155/2020/9456350)
Supplement: Supplementary Materials — Table S1: the information of the tested samples. Table S2: the specific composition for the different preparations. Table S3: precursor/product ion pairs and parameters for MRM of 21 components of XSHG. TABLE S4: relative distance for hematinic function evaluation between dose groups and control group from the PCA score plot of XSHG and decomposed recipes groups. Table S5: calibration curves, correlation coefficients, linearity ranges, and LOD and LOQ of the 21 investigated components. Table S6: precision, repeatability, stability, recovery, and the matrix effects of the 21 investigated compounds. Table S7: amounts (mg) of the aromatic acids and phthalides in the single herbs of Danggui and Chuanxiong. [file 9456350.f1.docx]

**Supporting Information**

**Supporting information:**

T_ABLE_ S1: The information of the tested samples.

T_ABLE_ S2: The specific composition for the different preparations.

T_ABLE_ S3: Precursor/product ion pairs and parameters for MRM of 21 components of XSHG.

T_ABLE_ S4: Relative distance for hematinic function evaluation between dose groups and control group from the PCA score plot of XSHG and decomposed recipes groups.

T_ABLE_ S5: Calibration curves, correlation coefficients, linearity ranges, LOD and LOQ of the 21 investigated components.

T_ABLE_ S6: Precision, repeatability, stability, recovery, and the matrix effects of the 21 investigated compounds.

T_ABLE_ S7: Amounts (mg) of the aromatic acids and phthalides in the single herbs of Danggui and Chuanxiong.

T_ABLE_ S1: The information of the tested samples.

| Sample number | | Species | Plant part | | | Collection region | Collection time |
| --- | --- | --- | --- | --- | --- | --- | --- |
| 1 | *Angelica sinensis* | | | Radix | Minxian, Gansu | | October 19, 2015 |
| 2 | *Leonurus japonicas* | | | Herb | Shantou, Guangdong | | October 08, 2015 |
| 3 | *Ligusticum chuanxiong* | | | Rhizome | Dujiangyan, Sichuan | | September 26, 2015 |
| 4 | *Prunus persica* | | | Seed | Lanzhou, Gansu | | August 15, 2015 |
| 5 | *Carthamus tinctorius* | | | Florets | Tacheng, Xinjiang | | August 04, 2015 |
| 6 | *Zingiber officinale* | | | Rhizome | Changshun, Guizhou | | November 23, 2015 |
| 7 | *Glycyrrhiza uralensis* | | | Radix | Yancheng, Ningxia | | October 18, 2015 |

T_ABLE_ S2: The specific composition for the different preparations.

| Samples | DG (g) | YMC (g) | CX (g) | TR (g) | HH (g) | JT (g) | ZGC (g) |
| --- | --- | --- | --- | --- | --- | --- | --- |
| XSHG | 240 | 300 | 90 | 24 | 15 | 15 | 15 |
| DY | − | − | 90 | 24 | 15 | 15 | 15 |
| DC | − | 300 | − | 24 | 15 | 15 | 15 |
| DT | − | 300 | 90 | − | 15 | 15 | 15 |
| DH | − | 300 | 90 | 24 | − | 15 | 15 |
| DJ | − | 300 | 90 | 24 | 15 | − | 15 |
| DZ | − | 300 | 90 | 24 | 15 | 15 | − |

T_ABLE_ S3: Precursor/product ion pairs and parameters for MRM of 21 components of XSHG.

| Analytes | t_R_ (min) | [M+H]^+^ (m/z) | [M−H]^−^ (m/z) | MRM transitions/SIM | Cone voltage (V) | Collision energy (eV) |
| --- | --- | --- | --- | --- | --- | --- |
| 1 trigonelline | 1.29 | 138 | ⎯ | 138 → 92 | 30 | 22 |
| 2 stachydrine hydrochloride | 2.13 | 127 | ⎯ | 127 → 110 | 22 | 16 |
| 3 protocatechuic acid | 4.36 | ⎯ | 153 | 153 → 109 | 20 | 14 |
| 4 hydroxysafflor yellow A | 5.79 | ⎯ | 611 | 611 → 491 | 32 | 26 |
| 5 chlorogenic acid | 5.87 | ⎯ | 353 | 353 → 191 | 18 | 20 |
| 6 amygdalin | 6.38 | ⎯ | 456 | 456 → 323 | 26 | 12 |
| 7 caffeic acid | 6.48 | ⎯ | 179 | 179 → 107 | 22 | 22 |
| 8 leonurine hydrochloride | 7.51 | 312 | ⎯ | 312 → 181 | 26 | 24 |
| 9 liquiritin | 8.11 | ⎯ | 417 | 417 → 255 | 26 | 16 |
| 10 ferulic acid | 8.24 | ⎯ | 193 | 193 → 134 | 24 | 14 |
| 11 zingerone | 9.58 | 195 | ⎯ | 195 → 137 | 6 | 12 |
| 12 isoliquiritoside | 9.83 | 419 | ⎯ | 419 → 137 | 18 | 38 |
| 13 senkyunolide I | 10.05 | 225 | ⎯ | 225 → 91 | 10 | 32 |
| 14 senkyunolide H | 10.43 | 225 | ⎯ | 225 → 91 | 10 | 32 |
| 15 liquiritigenin | 10.56 | 257 | ⎯ | 257 → 137 | 26 | 22 |
| 16 glycyrrhizic acid | 13.48 | ⎯ | 821 | 821 → 351 | 54 | 28 |
| 17 6-gingerol | 14.56 | 295 | ⎯ | 295 → 137 | 10 | 24 |
| 18 senkyunolide A | 15.13 | 193 | ⎯ | 193 → 137 | 18 | 14 |
| 19 ligustilide | 15.88 | 191 | ⎯ | 191 → 91 | 6 | 24 |
| 20 butylidenephthalide | 15.96 | 189 | ⎯ | 189 → 128 | 10 | 24 |
| 21 6-shogaol | 16.23 | 277 | ⎯ | 277 → 137 | 12 | 10 |

T_ABLE_ S4: Relative distance for hematinic function evaluation between dose groups and control group from the PCA score plot of XSHG and decomposed recipes groups.

| Control group (mean) | | Treated group | Distance (mean ± SD) |
| --- | --- | --- | --- |
| x-Axis | y-Axis |  |  |
| 5.44 | -2.59 | Model | 10.73 ± 0.58 |
|  |  | AJ | 5.58 ± 0.49^**^ |
|  |  | XSHG | 5.04 ± 0.29^**∆∆^ |
|  |  | DY | 8.18 ± 0.41^**∆∆^ |
|  |  | DC | 7.67 ± 0.37^**∆∆^ |
|  |  | DT | 6.39 ± 0.48^**∆∆^ |
|  |  | DH | 7.32 ± 0.64^**∆∆^ |
|  |  | DJ | 5.84 ± 0.54^**∆^ |
|  |  | DZ | 7.11 ± 0.35^**∆∆^ |

^*^ *P* < 0.05, ^**^ *P* < 0.01 vs distance of model group; ^∆^ *P* < 0.05, ^∆∆^ *P* < 0.01 vs distance of XSHG group.

T_ABLE_ S5: Calibration curves, correlation coefficients, linearity ranges, LOD and LOQ of the 21 investigated components.

| Analytes | Calibration curves | *r*^2^ | Linear range (μg/mL) | LOQ (ng/mL) | LOD (ng/mL) |  |
| --- | --- | --- | --- | --- | --- | --- |
| 1 trigonelline | | y = 1098.97x + 42.813 | 0.9995 | 0.07172 ~ 18.360 | 16.93 | 5.98 |
| 2 stachydrine hydrochloride | | y = 1159.68x + 21.389 | 0.9992 | 0.3035 ~ 77.707 | 9.60 | 3.23 |
| 3 protocatechuic acid | | y = 24.543x − 0.899 | 0.9996 | 0.01781 ~ 2.280 | 16.43 | 4.86 |
| 4 hydroxysafflor yellow A | | y = 196.43x + 23.571 | 0.9985 | 0.05563 ~ 56.958 | 5.63 | 1.78 |
| 5 chlorogenic acid | | y = 1016.21x − 40.858 | 0.9997 | 0.055125 ~ 7.056 | 9.06 | 2.94 |
| 6 amygdalin | | y = 584.20x + 21.541 | 0.9998 | 0.05456 ~ 28.162 | 3.52 | 1.17 |
| 7 caffeic acid | | y = 71.387x + 15.961 | 0.9996 | 0.05391 ~ 13.800 | 6.41 | 2.08 |
| 8 leonurine hydrochloride | | y = 20794.03x − 756.426 | 0.9989 | 0.09176 ~ 23.490 | 2.53 | 0.83 |
| 9 liquiritin | | y = 3141.53x − 52.988 | 0.9937 | 0.05513 ~ 7.056 | 5.28 | 1.78 |
| 10 ferulic acid | | y = 3303.11x + 1030.310 | 0.9988 | 0.04566 ~ 22.169 | 7.78 | 2.49 |
| 11 zingerone | | y = 572.52x − 21.386 | 0.9997 | 0.05813 ~ 3.720 | 10.84 | 3.63 |
| 12 isoliquiritoside | | y = 6430.92x − 16.706 | 0.9981 | 0.00305~ 0.391 | 11.53 | 3.81 |
| 13 senkyunolide I | | y = 2568.13x − 159.251 | 0.9999 | 0.05100 ~ 13.056 | 15.23 | 5.06 |
| 14 senkyunolide H | | y = 880.42x − 28.261 | 0.9979 | 0.05219 ~ 13.363 | 30.58 | 9.89 |
| 15 liquiritigenin | | y = 6113.39x − 53.099 | 0.9993 | 0.00951~ 1.216 | 7.58 | 2.52 |
| 16 glycyrrhizic acid | | y = 952.42x − 106.676 | 0.9973 | 0.09603 ~ 24.584 | 23.47 | 7.62 |
| 17 6-gingerol | | y = 424.72x − 22.454 | 0.9996 | 0.05963 ~ 7.632 | 26.49 | 8.76 |
| 18 senkyunolide A | | y = 8518.97x + 692.812 | 0.9993 | 0.04716 ~ 15.090 | 1.96 | 0.62 |
| 19 ligustilide | | y = 1036.43x + 178.561 | 0.9992 | 0.06818 ~ 34.910 | 17.69 | 5.46 |
| 20 butylidenephthalide | | y = 4277.12x + 122.270 | 0.9969 | 0.03445 ~ 1.0850 | 4.68 | 1.53 |
| 21 6-shogaol | | y = 10563.79x + 36.683 | 0.9982 | 0.00521 ~ 0.333 | 4.92 | 1.61 |

T_ABLE_ S6: Precision, repeatability, stability, recovery, and the matrix effects of the 21 investigated compounds.

| Analytes | precision (RSD, %; *n* = 6) | | Repeatability  (RSD, %; *n* = 6) | Stability  (RSD, %; *n* = 6) | Recovery (%, *n* = 3) | | matrix effects |
| --- | --- | --- | --- | --- | --- | --- | --- |
|  | intraday | interday |  |  | Mean | RSD |  |
| 1 trigonelline | 1.89 | 3.32 | 4.86 | 2.67 | 96.52 | 3.35 | 0.92 |
| 2 stachydrine hydrochloride | 2.65 | 4.06 | 2.17 | 3.71 | 97.45 | 2.81 | 0.99 |
| 3 protocatechuic acid | 1.58 | 4.38 | 3.32 | 2.34 | 97.92 | 4.23 | 1.05 |
| 4 hydroxysafflor yellow A | 3.26 | 3.94 | 3.51 | 1.83 | 96.96 | 2.58 | 0.93 |
| 5 chlorogenic acid | 1.95 | 2.65 | 3.06 | 3.87 | 98.41 | 3.32 | 0.96 |
| 6 amygdalin | 2.82 | 2.94 | 2.87 | 3.59 | 97.16 | 3.26 | 1.07 |
| 7 caffeic acid | 2.67 | 2.45 | 3.04 | 3.62 | 102.37 | 4.66 | 0.96 |
| 8 leonurine hydrochloride | 1.49 | 1.72 | 3.89 | 1.37 | 97.86 | 2.48 | 1.03 |
| 9 liquiritin | 1.47 | 2.32 | 1.85 | 2.53 | 96.03 | 2.51 | 0.93 |
| 10 ferulic acid | 1.87 | 2.58 | 4.23 | 2.39 | 94.79 | 3.27 | 0.92 |
| 11 zingerone | 2.51 | 2.42 | 2.96 | 3.63 | 96.52 | 1.54 | 0.96 |
| 12 isoliquiritoside | 1.61 | 2.35 | 2.74 | 3.16 | 98.32 | 3.43 | 0.91 |
| 13 senkyunolide I | 1.79 | 1.87 | 2.38 | 1.67 | 98.29 | 2.48 | 0.98 |
| 14 senkyunolide H | 1.96 | 2.91 | 3.70 | 2.58 | 99.86 | 4.35 | 0.93 |
| 15 liquiritigenin | 2.12 | 2.55 | 2.46 | 4.59 | 95.48 | 1.31 | 1.01 |
| 16 glycyrrhizic acid | 1.06 | 1.43 | 2.58 | 2.83 | 102.76 | 4.08 | 0.94 |
| 17 6-gingerol | 2.29 | 2.77 | 3.52 | 3.96 | 98.95 | 2.67 | 0.98 |
| 18 senkyunolide A | 2.51 | 2.94 | 3.27 | 3.84 | 95.87 | 4.78 | 0.96 |
| 19 ligustilide | 2.93 | 3.09 | 3.58 | 3.42 | 98.98 | 2.83 | 1.04 |
| 20 butylidenephthalide | 2.34 | 2.61 | 3.40 | 2.48 | 100.52 | 2.69 | 0.99 |
| 21 6-shogaol | 2.45 | 3.87 | 3.93 | 4.26 | 99.37 | 2.88 | 0.93 |

T_ABLE_ S7: Amounts (mg) of the aromatic acids and phthalides in the single herbs of Danggui and Chuanxiong.

| Sample | Analyte / amounts (mg, *n* = 6) | | | | | | | | |
| --- | --- | --- | --- | --- | --- | --- | --- | --- | --- |
|  | 3 | 5 | 7 | 10 | 13 | 14 | 18 | 19 | 20 |
| DG (240 g) | 20.41 ± 0.61 | 46.32 ± 3.18 | 19.68 ± 0.43 | 157.20 ± 10.47 | 73.21 ± 5.48 | 49.92 ± 2.19 | 344.40 ± 10.34 | 453.36 ± 18.93 | 23.04 ± 0.57 |
| CX (90 g) | 20.52 ± 1.13 | 58.14 ± 2.29 | 17.46 ± 1.05 | 70.65 ± 3.42 | 59.31 ± 4.52 | 32.37 ± 1.44 | 226.98 ± 12.66 | 242.28 ± 13.07 | 27.27 ± 1.43 |
| proportionality coefficient of CX | 0.501 | 0.557 | 0.470 | 0.310 | 0.448 | 0.358 | 0.397 | 0.348 | 0.542 |

Proportionality coefficient of the analytes in CX could be calculated by the equation: (Amount in CX) / (Amount_._ in DG + Amount_._ in CX).
